# Configurational-based institutional analysis: Unbundling the multi-dimensional state fragility construct

**DOI:** 10.1016/j.mex.2021.101209

**Published:** 2021-01-19

**Authors:** Joshua K. Ault, Andrew Spicer

**Affiliations:** a Thunderbird School of Global Management, Arizona State University, MC 1221, 400 E Van Buren, Ste 800, Phoenix, AZ 85004-2262 USA; b Darla Moore School of Business, Sonoco International Business Department, University of South Carolina, 1014 Greene St, Columbia, SC 29208 USA

**Keywords:** State fragility, Developing countries, Comparative institutional analysis, Configurational-based research

## Abstract

To guide researchers interested in replicating our configurational approach to comparative institutional analysis, we report on a methodology that builds from existing state fragility indices to differentiate between types of state fragility in 189 countries across the world. We first present the steps we used to identify three constituent dimensions of state fragility. We then report how we configured these first-order institutional measures to build a large-N, fuzzy set qualitative set analysis (fs/QCA) of variation in the degree and type of informal economic sectors found across the world. Our discussion of the methodology shows that:

• Configurational-based analysis requires unbundling aggregate measures into constituent dimensions as a first step to analyzing possible systemic interactions.
• Configurational-based analysis recognizes sources of cross-national diversity that aggregated or single dimensional metrics are unable to fully capture.

## Graphical abstract

**Figure:**
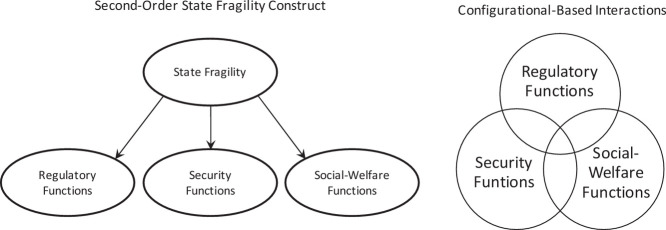

**Table:** Specifications Table

Subject Areas:	Economics, Business, Political Science
More Specific Subject Areas:	International Development, Comparative Entrepreneurship, Comparative Institutional Analysis
Method Name:	Configurational-Based Analysis of State Fragility Interactions
Names and References of Original Method	Ault, J. K., & Spicer, A. 2020. State Fragility as a Multi-Dimensional Construct for International Entrepreneurship Research and Practice. ***Asia Pacific Journal of Management*,** 37(4): 981-1011.
	Ault, J. K., & Spicer A. Forthcoming. The Formal Institutional Context of Informal Entrepreneurship: A Cross-National, Configurational-Based Perspective. ***Research Policy***.
Resource Availability:	https://www.joshuakault.com/ault-spicer-state-fragility-index

Building on a long tradition in political science, the state fragility literature starts with the observation that an important source of cross-national variation rests in the state's capabilities to implement laws, policies, and programs. For instance, Huntington [[20]: 1] observes that: “The most important political distinction among countries concerns not their form of government but their degree of government,” and therefore a state's ability to provide basic government functions needs to be empirically tested and measured rather than assumed (see also [11]). The subsequent conceptualization and measurement of state fragility as a multidimensional construct has emerged as one effort to empirically compare political systems based on core state capabilities rather than regime types or formal policies [13,^17^,27].

While empirical measures of state fragility have historically relied on a single score at an aggregate level of analysis, researchers in this tradition have recently called for more nuanced empirical work that distinguishes between types rather than only degree of state capabilities [1,^3^,^14^,15]. To illustrate, suppose the government in one country has difficulty in implementing a rule of law; a second has trouble in providing basic social welfare services; and a third is unable to provide peace and security. If measured along a single dimension of overall state fragility, all three states would receive a similarly low score when compared to an ideal-typical developed country (such as Denmark or Norway) where the state possesses strong capabilities across all dimensions. However, such an aggregation strategy may mask the fact that each state is weak in its own way and therefore significantly different from the others [1].

In this paper, we describe the methodology we developed to implement a configurational-based institutional analysis to capture cross-national differences in the kind, rather than only the degree, of state capabilities [1,2]. Our aim here is to guide researchers who wish to replicate our configurational-based methodology to analyze other types of outcomes or other time periods than our original analyses. This paper provides an overview of the extended methodology that began with a confirmatory factor analysis (CFA) that identified three primary underlying dimensions of state fragility [1] and then extended to the development of a large-N, fuzzy set qualitative comparative analysis (or large-N fs/QCA; see [16]) that examined possible systemic interactions across these dimensions [2]. As the fuzzy set analysis would not have been possible to conduct without our preliminary empirical work, we describe here the entire methodology in an interconnected narrative that connects previous stand-alone work.

Moreover, we include additional information not found in our other papers to aid in the details of replication. For instance, we include the Stata coding we used to estimate our confirmatory factor analysis. We also include democracy as a separate dimension of a country's political system in the factor analysis reported here, which extends the analyses presented in Ault and Spicer [1]. Finally, we provide a truth table, an intermediate step of fs/QCA that we did not have space to report or evaluate in Ault and Spicer [[2] forthcoming]. The truth table provides a further illustration of the benefits of a configurational-based institutional analysis to identify significant sources of cross-national diversity that aggregated or single dimensional metrics are unable to fully recognize.

## Constituent dimensions of state fragility

In Ault and Spicer [1], we conducted a confirmatory factor analysis (CFA) on a large set of indicators found in two existing state fragility indexes: the Polity IV State Fragility Index [26] and the Brooking Institute's Index of State Weakness in the Developing World [34]. We chose these two sources because they adopt a similar functional-based approach that divides state capabilities into four primary dimensions: (1) political; (2) social-welfare; (2) security; and (4) economic. In these data sets, the “political” dimension refers to all state functions that create an effective, independent, and responsive system of governance (e.g., rule of law, control of corruption); the “social-welfare” dimension includes state functions related to basic human needs (e.g., education, healthcare); the “security” dimension refers to functions aimed at maintaining the government's monopoly over violence (e.g., policing, border security); and the “economic” dimension includes functions that provide a stable economic environment, private sector development, and sustainable growth (e.g., limited inflation, income equality).

While these two sources adopt similar categories for their state fragility index, they chose different indicators to measure each dimension. To deal with this lack of congruence between the indexes, we collected all 22 of the publicly-available indicators identified in each index. While Rice and Patrick [34] only provided scores for the single year of 2008, we collected data from the underlying sources for the year 2016.^1^ We began our analysis with all 193 member-states of the United Nations [36]. Of these, 4 (Bahamas, Brunei Darussalam, Monaco, San Marino) did not provide sufficient data and were dropped. Our final sample was thus 189 countries. To ensure commensurability, we coded all indicators such that a higher score indicated greater state fragility.

In the first iteration of the CFA, we loaded each indicator on the same first-order dimensions identified in Marshall and Cole [26] and Rice and Patrick [34] using the “sem” command in Stata. Next, we evaluated the fit statistics using the “estat gof” command and the modification indices (MI) using the “estat mindices” command. MI is an estimate of the amount by which the fit statistics would improve if correlations between residuals are added and freely estimated. For instance, the residuals for access to clean water and life expectancy were found to correlate, but statistical software packages would not automatically estimate these parameters. We thus used the “cov” option to specify that these, and other variables with similarly correlated error terms, should be allowed to covary.

In subsequent iterations of the CFA, we first removed or shifted any variables from the analysis that did not load on the predicted latent factor at confidence levels greater than 0.95. Table 1 reports the final standardized factor loadings of this analysis. Results show that the political factor includes rule of law, control of corruption, government effectiveness, and regulatory quality; social-welfare includes child mortality, access to water, life expectancy, and human development; security includes political stability and political terror.

**Table 1:** Best Fit Results of Confirmatory Factor Analysis of State Fragility and Democracy Indicator.

Variable	Std. Coef.	*p*-value
**Regulatory**		
Government Effectiveness	0.98	0.00 ***
Regulatory Quality	0.96	0.00 ***
Rule of Law	0.97	0.00 ***
Control of Corruption	0.93	0.00 ***
**Social Welfare**		
Child Mortality	0.90	0.00 ***
Access to Clean Water	0.80	0.00 ***
Life Expectancy	0.92	0.00 ***
Human Development Index	1.00	0.00 ***
**Security**		
Political Stability/Absence of Violence	0.92	0.00 ***
Political Terror	0.90	0.00 ***
**Democracy**		
Democracy	0.96	0.00 ***
Voice & Accountability	1.00	0.00 ***
Electoral Freedom	0.99	0.00 ***
**Covariance**		
Regulatory/Welfare	-0.84	
Regulatory/Security	0.78	
Regulatory/Democracy	0.74	
Welfare/Security	-0.64	
Welfare/Democracy	-0.58	
Security/Democracy	0.71	
**Fit Statistics**		
*X^2^*	248.30***	
RMSEA	0.15	
CFI	0.95	
TLI	0.94	
SRMR	0.04	
CD	1.00	

In this factor analysis, we found that undernourishment, primary school completion rate, major episodes of political violence, coup d’états, GDP growth rate, GDP per capita, Gini, and inflation did not load on any state fragility factor. The last four of these measures had been placed within an economic dimension in the Marshall and Cole [26] and Rice and Patrick [34] state fragility indexes. However, since we did not find empirical support for a separate economic dimension, we dropped these indicators from our final index. Similarly, we found that the Kaufmann et al. [23] measure of a state's “regulatory quality” did not fit within an economic dimension of state fragility, as suggested in the Rice and Patrick [34] Index, but did fit within the political dimension (*p* = 0.00). We therefore moved this measure into a country's political score.

Table 1 adds additional information to the results of a similar factor analysis reported in Ault and Spicer [1]. First, our earlier factor analysis discarded the three measures of democracy (democracy, electoral freedom, voice & accountability). While the existing indexes identified these measures as indicators of the political dimension of state fragility, we found that they did not load on this factor and thus did not include these measures in our final analysis. However, in a separate follow-up confirmatory factor analysis conducted in Ault and Spicer [[2] forthcoming], we found that these indicators were highly correlated among themselves, and loaded on their own factor. In contrast to the factor analysis reported in Ault and Spicer [1], Table 1 includes “democracy” as a separate construct. This democracy factor reflects a country's regime type, while the state fragility dimensions measure the state's ability to implement and enforce core functions across its regulatory, social-welfare, and security functions.

Second, the inclusion of a separate democracy factor led us to change the name of the “Political “dimension of the state fragility construct to the “Regulatory” dimension as a way to reduce confusion. While both democracy and regulations are part of a country's political domain, “democracy refers to citizen participation in lawmaker selection” while “regulatory fragility deals with the state's inability to implement its own laws and policies” ([2]: 19).

The next step in our confirmatory factor analysis was to examine the modification indices produced using Stata's “estat mindices” command. In confirmatory factor analysis, modification indices are commonly used to determine how much the model fit would improve if the error terms of certain variables are allowed to covary [25]. This step indicated that the fit of our model could be improved by allowing (1) child mortality and life expectancy; (2) rule of law and corruption; and (3) political terror and democracy to covary. We made this change using the “cov” option of the “sem” command in Stata.

Table 1 presents the final fit statistics (*X^2^,* RMSEA, CFI, TLI, SRMR). While *X^2^* and RMSEA are typically reported, they are sensitive to sample size and are thus often not interpreted [21]. All other fit statistics suggest a strong fit of the final model.

Finally, we tested the discriminant validity of our dimensions. To do this, we took the two factors with the highest covariance (political and social-welfare) and loaded all 8 of their combined indicators onto a single factor. In this test, CFI, TLI, and SRMR all deteriorated past conventional cutoffs for acceptable fit. Moreover, the standardized factor loadings for nearly all variables previously included on the social-welfare dimension fell below 0.80, thus supporting discriminant validity: a state's regulatory, social-welfare, and security capabilities are related to one another but do not represent the same concept.

To convert the factor analysis into scores for comparative analysis, we took each of the 13 individual indicators that we initially found to load on the first-order factors of state fragility [1], plus the democracy indicators we identified through our subsequent analysis [2], and standardized them. As before, we used an inverted score for any measures where a higher score indicated lower fragility (e.g., access to clean water, life expectancy). Next, we aggregated the individual indicators for each first-order factor. To do this, we weighted our factors by first multiplying each indicator by its standardized factor loading and then took the average across the indicators that fit on the same factor. Finally, we again standardized each first-order factor to make them comparable to one another.

We then constructed a second-order state fragility factor by taking a straight average across the three first-order factors (regulatory, social-welfare, and security). In creating the second-order scores, we excluded the democracy factor, since the state fragility literature suggests that democracy belongs to a theoretically different construct [1,^13^,18]. The full set of country level scores are reported in Ault and Spicer [1], and are available at the website: https://www.joshuakault.com/ault-spicer-state-fragility-index.

## Configurational-based analysis

For the next step in our comparative institutional analysis, we took the underlying first-order dimensions of state fragility identified through our factor analysis (regulatory, social-welfare, security) and applied them as causal conditions in a large-N, fuzzy set qualitative comparative analysis (or large-N, fs/QCA; see [16]). We chose a large-N fs/QCA methodology because it allowed us to include many countries in our analysis without forcing us to make population-level assumptions of causal homogeneity across all country cases at the start of a study [2]. Contrary to small-n fs/QCA, where relevant causal conditions can be identified through inductive reasoning within the analysis itself, large-N fs/QCA requires the set of relevant causal conditions to be identified deductively before the analysis begins [16,22]. The critical input from our initial empirical work in Ault and Spicer [1] was therefore to systemically identify the primary constituent parts of the underlying state fragility construct to permit a configurational-based analysis through a large-N, fs/QCA methodology.

The focal outcome condition for our analysis was variation in the degree and type of informal economic sectors across the world. While the data are explained more fully in Ault and Spicer [[2] forthcoming], a basic description is necessary to understand the analysis that follows. To develop national measures of a country's informal economic sector, we used the estimations available in Loayza's [24] “Toolkit for Informality Scenario Analysis” of the number of all workers that do not possess a formal labor contract. Globally, Loayza [24] estimates that two-thirds of all workers (2 billion individuals) operate in the informal sector. Moreover, this dataset breaks down the overall size of the informal sector into two different types of activity. First, Loayza [24] estimates that 71.5% (1.43 billion) informal workers own rudimentary enterprises that pursue subsistence-oriented goals and hire, if at all, from within the owner's immediate family. Second, he estimates that the remaining 28.5% (570 million) work in firms that hire off the books but nonetheless seek opportunities to grow beyond small, owner-operated ventures [2].

Loayza [24] provides estimates for each of these outcomes for 149 countries, based on 2016 data, which we use to build a set of fs/QCA models that explain three distinct outcomes that have recently gained attention in the field of comparative entrepreneurship: (1) differences in the level of informal entrepreneurship across country contexts; (2) differences in the level of subsistence-oriented informal entrepreneurship across country contexts; and (3) differences in the level of growth-oriented informal entrepreneurship across country contexts.

For our causal conditions, we included each of the three state fragility dimensions created through the confirmatory factor analysis described above, plus democracy. To ease the interpretation of our results, we inverted each of these measures, so that a present condition would indicate state capability, rather than fragility. We also changed the name of the political dimension to the “regulatory” dimension to avoid confusion with democracy. Additionally, since we did not find support for an economic dimension, but were still interested in how income levels might impact entrepreneurship in the informal sector, we included the [38] measure for GNI per capita, Atlas method in the model [38]. Finally, we added a measure for each country's population to assess the degree to which large emerging markets represent a distinct country subpopulation. Our final sample included all countries found in: (1) the state fragility dataset we created through the factor analysis described above; (2) the Loayza [24] dataset; and (3) the World Bank's [38] World Development Indicators. In all 138 country cases were found in all three.

To conduct the fs/QCA, we used Ragin and Davey's [33] fs/QCA software and followed the steps identified by Ragin [31,32] and further developed by others (e.g., [8,^9^,^16^,^22^,^30^,37]). We first calibrated the data into membership scores for each case according to its deviation from set anchors for full membership, full non-membership, and a crossover point. For large-N fs/QCA, authors recommend using either external benchmarks [30] or, when external benchmarks are unavailable, using the median for the crossover point and the 25^th^ and 75^th^ percentiles as the lower and upper thresholds [16]. Following this guideline, we relied on external benchmarks to anchor our calibration for GNI per capita. Specifically, we follow Ragin [[32]: 90] and set the anchor for “full membership” at $20,000. To calibrate the other thresholds for this condition, we followed the World Bank's [39] cutoffs for high-income ($12,476), upper-middle income ($4,036), and lower-middle income ($1,025). Given that external benchmarks were unavailable for our remaining measures, we used the median as the crossover and the 25^th^ and 75^th^ percentiles as the lower and upper thresholds.

After calibrating our data, we then conducted necessity tests on each of the causal conditions, and their negations, individually to identify any that must be present, or must be absent, for the outcome to occur. Following the precedent in the literature, we determined that any causal attribute with a consistency greater than 0.85 and coverage greater than 0.50 would pass the necessity test [19].

Next, we created a Truth Table, or a data matrix that lists all logically possible combinations of causal conditions associated with the outcome [4,5]. We then reduced the Truth Table according to (1) a minimum number of cases required in a given configuration for a solution to be considered; and (2) a lowest acceptable consistency level consistency and/or “proportional reduction in inconsistency” (PRI) level [4,9]. Consistency refers to the accuracy of the statement “if X, then Y” and is calculated as Σmin(x_i_,y_i_)/Σx_i_, where X signifies the predictor configuration, Y signifies the outcome set, x_i_ stands for each case's membership in configuration X, and y_i_ represents each case's membership in configuration Y [29]. Setting a PRI threshold helps avoid the simultaneous inclusion of configurations that are found both in the outcome and its absence. For our analysis, we retained any configurations associated with at least two country cases and identified a consistency threshold at 0.85 and a PRI threshold at 0.75.

We present the reduced Truth Table in Table 2. As shown here, our analysis produced 18 different country-case configurations. In this version of the reduced Truth Table, an “X” means that the set of countries in the configuration all sit above the cross-over point on that condition (which is the global median for all measures except GNI per capita). As shown in the first column, we grouped the configurations together in the table according to the conjoint presence or absence of the three state fragility (or in this analysis, state capacity) factors; we classified configurations that possess all three capacities as fully-strong, those that lack all three conditions as fully-weak, and those with a mix of presence and absence of the three condition as hybrid. The table also includes the number of countries that the software identified as greater than 0.50 membership in the specified configuration. Finally, the last column lists some exemplary countries that the software identified as possessing the strongest membership in that configuration.

**Table 2:** Reduced Truth Table.

Country Classification	Attribute						No. of Cases	Exemplary Countries
	Population	GNI per Cap.	Democracy	Regulatory	Social Welfare	Security		
**Fully Strong**		X	X	X	X	X	20	Norway, Denmark, Ireland
	X	X	X	X	X	X	13	U.S., Germany, Japan
			X	X	X	X	8	Bulgaria, Costa Rica, Croatia
		X		X	X	X	3	Singapore, Qatar, Oman

**Hybrid**						X	5	Laos, Moldova, Fiji
	X			X	X		4	Turkey, China, Thailand
			X			X	4	Paraguay, Mongolia, Guyana
	X				X		3	Russia, Iran
	X		X		X		3	Mexico, Brazil, Tunisia
	X					X	3	Morocco, Cambodia, Guinea
	X		X	X			3	South Africa, Colombia, India
					X		2	Lebanon, Belarus
		X		X	X		2	Kuwait, Bahrain
			X	X		X	2	Botswana, Namibia

**Fully Weak**	X						25	Pakistan, Nigeria, Cameroon
							10	Congo, Swaziland, Kyrgyzstan
	X		X				2	Philippines, Indonesia
			X				2	Lesotho, El Salvador

*Note:* X means that the condition is present in that configuration, while a blank space indicates that the condition is absent.

Forty-four countries fell into the category we refer to as “fully strong” states, since all these countries received scores that indicated the presence of strong capabilities across all domains. In this subset, countries differ along population, income, and democracy attributes rather than state capabilities. At the opposite extreme, the Truth Table identified 39 countries that we label “fully weak” states, since these states all fall below the global median across the three dimensions of state capability. In the middle of the Truth Table sit 31 countries that we label “hybrid institutional configurations,” as they possess strong capabilities in some domains and weak capabilities in others.

We then used the Quine-McCluskey algorithm, which is the default option in Ragin and Davey's [33] fs/QCA software, to logically reduce the numerous complex causal conditions into a simplified set of pathways that lead to the designated outcome [8,22]. This step distinguishes between core and peripheral attributes of configurational-based results. Core attributes are those for which the evidence indicates a strong causal relationship with the outcome, while peripheral attributes are those for which the evidence for a causal relationship with the outcome is weaker ([8]: 394).

The substantive results of the fs/QCA are reported in full in Ault and Spicer [[2] forthcoming]. However, we make some broad observations here to illustrate the benefits of analyzing state fragility as a configurational-based causal condition. Table 3 reports the results associated with large and small subsistence-oriented informal sectors, and Table 4 reports the results associated with large and small growth-oriented informal sectors. Consistent with other fs/QCA authors (e.g., [4,^6^,22]), we denote the presence of a condition within a configurational pathway with a black circle (“”) and the absence of a condition along a pathway with a slashed circle (“∅”). A blank space with no circle denotes a condition that may be either present or absent in a particular configuration. The size of the circles indicates whether the element is core or peripheral; a large circle (whether black or slashed) denotes a core condition, while a small circle indicates a peripheral condition. Many of the pathways identified in the Tables share core conditions, and only differ in their peripheral conditions. We thus group these pathways together and number them 1a, 1b, etc. Finally, we indicate which conditions passed the necessity tests at the bottom of each table.

**Table 3:** Pathways to Large and Small Subsistence-Oriented Informal Sectors.

	Large				Small	
	Poor, Welfare-Fragile States				Democratic, Fully-Strong States	
	1a	1b	1c	1d	2a	2b
**Population**						
**GNI per Capita**						
**Democracy**						
**Regulatory**						
**Social-Welfare**						
**Security**						
**Raw Coverage**	0.63	0.19	0.62	0.38	0.42	0.52
**Unique Coverage**	0.02	0.02	0.02	0.01	0.08	0.19
**Consistency**	0.90	0.93	0.89	0.89	0.97	1.00
**Solution Coverage**	0.80				0.61	
**Solution Consistency**	0.89				0.98	
Necessary Conditions(s)*	Low Welfare Capability; Low GNI per Capita				High Welfare Capability	

Legend:  Core Condition Present;  Core Condition Absent;  Peripheral Condition Present;  Peripheral Condition Absent

* Causal attributes with consistency > 0.85 and coverage > 0.50.

**Table 4:** Pathways to Large and Small Growth-Oriented Informal Sectors.

	Large					Small
	Large Emerging Economies		Autocratic, Regulatory-Capable States	Regulatory-Capable, Welfare-Fragile States		Rich Democracies
	6a	6b	7	8a	8b	9
**Population**						
**GNI per Capita**						
**Democracy**						
**Regulatory**						
**Social-Welfare**						
**Security**						
**Raw Coverage**	0.17	0.18	0.10	0.11	0.11	0.49
**Unique Coverage**	0.01	0.02	0.06	0.03	0.06	0.49
**Consistency**	0.84	0.85	0.91	0.86	0.85	0.90
**Solution Coverage**	0.36					0.49
**Solution Consistency**	0.85					0.90
Necessary Conditions(s)*	None					None

Legend:  Core Condition Present;  Core Condition Absent;  Peripheral Condition Present;  Peripheral Condition Absent

⁎ Causal attributes with consistency > 0.85 and coverage > 0.50.

Overall, as further described in Ault and Spicer [[2] forthcoming], we find that countries with hybrid institutional characteristics defined by a mixture of strength in some domains and weakness in others do not sit halfway along a linear continuum between fully strong and fully weak states, but instead represent an independent set of institutional conditions that requires separate analysis in their own right. Each of the configurations associated with large subsistence-oriented informal sectors are defined by weakness across the dimensions of state capacity (see Table 3), while each of the configurations associated with large growth-oriented informal sectors are defined by a hybrid of strengths on some dimensions and weaknesses on others (see Table 4). While the former is closely associated with the “fully weak” states previously illustrated in the Truth Table, the latter is associated with those that possess “hybrid” institutional characteristics. Therefore, we propose that the formal institutional-based conditions that differentiate between types of informal sectors are best identified by the conjoint mixture of strength and weakness of state capabilities across multiple domains, rather than by uniform weakness, or voids, along all state functions.

## Conclusion

In summary, the results of both the confirmatory factor analysis (CFA) and fuzzy set qualitative comparative analyses (fs/QCA) demonstrate the utility of a configurational-based state fragility framework for country-level comparative institutional analysis. In the confirmatory factor analysis, we found support for the presence of three primary dimensions of state fragility (regulatory, social-welfare, and security) that relate to one another but remain discrete constructs. A country-level comparative analysis of informal entrepreneurial activity using fs/QCA further illustrated that different configurations of the first-order factors of state fragility, rather than a single variable or aggregated measure, best explains observed outcomes.

## Declaration of Competing Interest

The authors certify that they have no affiliation with or involvement with any organization or entity with any financial interest (such as honoraria; educational grants; participation in speakers’ bureaus; membership, employment, consultancies, stock ownership, or other equity interest; and expert testimony or patent-licensing arrangements), or non-financial interest (such as personal or professional relationships, affiliations, knowledge or beliefs) in the subject matter or materials discussed in this manuscript.

## Footnotes

1 The indicators, as grouped by their data sources, are: **(1) Kaufmann, Kraay, and Mastruzzi**[23]: Voice and Accountability, Political Stability and Absence of Violence, Government Effectiveness, Regulatory Quality, Rule of Law, Control of Corruption; **(2) World Bank**[38]**:** Mortality Rate Under 5, Primary School Completion Rate, Prevalence of Undernourishment, Population with Access to Improved Water, Life Expectancy, GNI per Capita, GDP Growth Rate, Gini Index, Inflation Rate **(3) Marshall, Gurr, and Jaggers**[28]**:** Major Episodes of Political Violence, Coup d’états, Regime Type; **(4) Gibney et al.**[12]: The Political Terror Scale; **(5) Freedom House**[10]**:** Electoral Freedom; **(6) UNDP**[35]**:** Human Development Index; (**7) EIU**[7]**:** Democracy Index
